# Using pooled data to estimate variance components and breeding values for traits affected by social interactions

**DOI:** 10.1186/1297-9686-45-27

**Published:** 2013-07-26

**Authors:** Katrijn Peeters, Esther Dorien Ellen, Piter Bijma

**Affiliations:** 1 Animal Breeding and Genomics Centre, Wageningen University, P.O. Box 338, 6700 AH Wageningen, The Netherlands

## Abstract

**Background:**

Through social interactions, individuals affect one another’s phenotype. In such cases, an individual’s phenotype is affected by the direct (genetic) effect of the individual itself and the indirect (genetic) effects of the group mates. Using data on individual phenotypes, direct and indirect genetic (co)variances can be estimated. Together, they compose the total genetic variance that determines a population’s potential to respond to selection. However, it can be difficult or expensive to obtain individual phenotypes. Phenotypes on traits such as egg production and feed intake are, therefore, often collected on group level. In this study, we investigated whether direct, indirect and total genetic variances, and breeding values can be estimated from pooled data (pooled by group). In addition, we determined the optimal group composition, *i.e.* the optimal number of families represented in a group to minimise the standard error of the estimates.

**Methods:**

This study was performed in three steps. First, all research questions were answered by theoretical derivations. Second, a simulation study was conducted to investigate the estimation of variance components and optimal group composition. Third, individual and pooled survival records on 12 944 purebred laying hens were analysed to investigate the estimation of breeding values and response to selection.

**Results:**

Through theoretical derivations and simulations, we showed that the total genetic variance can be estimated from pooled data, but the underlying direct and indirect genetic (co)variances cannot. Moreover, we showed that the most accurate estimates are obtained when group members belong to the same family. Additional theoretical derivations and data analyses on survival records showed that the total genetic variance and breeding values can be estimated from pooled data. Moreover, the correlation between the estimated total breeding values obtained from individual and pooled data was surprisingly close to one. This indicates that, for survival in purebred laying hens, loss in response to selection will be small when using pooled instead of individual data.

**Conclusions:**

Using pooled data, the total genetic variance and breeding values can be estimated, but the underlying genetic components cannot. The most accurate estimates are obtained when group members belong to the same family.

## Background

Group housing is common practice in most livestock farming systems. Previous studies have shown that group-housed animals can substantially affect one another’s phenotype through social interactions [1-9]. The heritable effect of an individual on its own phenotype is known as the direct genetic effect, while the heritable effect of an individual on the phenotype of a group mate is known as the social, associative or indirect genetic effect [10-14]. Both direct and indirect genetic effects determine a population’s potential to respond to selection, *i.e.* the total genetic variance [2,10-14]. Selection experiments in laying hens and quail [1,2,9], and variance component estimates in laying hens, quail, beef cattle and pigs [3-9] have shown that indirect genetic effects can contribute substantially to the total genetic variation in agricultural populations.

Direct, indirect and total genetic variances can be estimated from individual data. However, it can be difficult or expensive to obtain individual phenotypes on certain traits, *e.g.* egg production and feed intake. Alternatively, data can be obtained on group level, resulting in pooled records. However, pooling data reduces the number of data points. Moreover, multiple animals influence each data point, increasing the complexity of the data. Although there is an obvious loss of power, previous studies have shown that pooled data can be used to estimate direct genetic variances for traits not affected by social interactions [15-17]. However, with social interactions, indirect genetic effects emerge and the complexity of the data increases further. It is unclear whether pooled data are still informative in these situations. Therefore, the main objective of this study was to determine whether pooled data can be used to estimate direct, indirect and total genetic variances, and breeding values for traits affected by social interactions. In addition, optimal group composition was determined, *i.e.* the optimal number of families represented in a group to minimise the standard error of the estimates.

## Methods

This study was performed in three steps. First, all research questions were answered by theoretical derivations. Second, a simulation study was conducted to investigate the estimation of variance components and optimal group composition. Third, individual and pooled survival records on 12 944 purebred laying hens were analysed to investigate the estimation of breeding values and response to selection.

Table 1 lists the main symbols and their meaning.

**Table 1:** Notation key

**Symbol**	**Meaning**
i – j	Focal individual - Group mates of the focal individual
*A*_D_	Direct genetic effect \ Direct breeding value
*A*_I_	Indirect genetic effect \ Indirect breeding value
*A*_T_	Total genetic effect \ Total breeding value
*E*_D_	Direct environmental effect
*E*_I_	Indirect environmental effect
$\sigma_{A_{D}}^{2}$	Direct genetic variance
$\sigma_{A_{\mathrm{DI}}}$	Direct–indirect genetic covariance
$\sigma_{A_{I}}^{2}$	Indirect genetic variance
$\sigma_{A_{T}}^{2}$	Total genetic variance
$\sigma_{\mathrm{Cage}}^{2}$	Cage variance
$\sigma_{E}^{2}$	Error variance
$\sigma_{P}^{2}$	Phenotypic variance
$\sigma_{E^{*}}^{2}$	Pooled error variance
$\sigma_{P^{*}}^{2}$	Pooled phenotypic variance
*h*^2^	Direct genetic variance relative to phenotypic variance \ Heritability
*T*^*2*^	Total genetic variance relative to phenotypic variance
$\sigma_{z}^{2}$	Full variance
$\sigma_{b}^{2}$	Between-family variance
$\sigma_{w}^{2}$	Within-family variance
*r*	Relatedness within a family
*N*	Number of families
*m*	Number of records per family
*o*	Family size
*n*	Group size
*^*	Hat, denotes estimated values

### Theory

#### Variance components and breeding value estimation

In this section, we examined whether direct, indirect and total genetic variances, and breeding values can be estimated from pooled data.

With social interactions, an individual phenotype consists of the direct genetic (*A*_D_) and environmental (*E*_D_) effects of the individual itself (*i*), and the indirect genetic (*A*_I_) and environmental (*E*_I_) effects of its group mates (*j*):

(1) $$P_{i}=A_{D_{i}}+E_{D_{i}}+\sum_{i\neq j}^{n-1}A_{I_{j}}+\sum_{i\neq j}^{n-1}E_{I_{j}},$$

where *n* is the number of individuals per group [11]. From an animal breeding perspective, the total breeding value (*A*_T_) is of interest because it determines total response to selection. An animal’s *A*_T_ consists of a direct and indirect component:

(2) $$A_{T_{i}}=A_{D_{i}}+\left(n‒1\right)A_{I_{i}},$$

where *A*_D_ is expressed in the phenotype of the animal itself and *A*_I_ is expressed in the phenotype of each group mate.

A pooled record (P^*^) consists of the individual phenotypes of all group members (*k*):

(3) $$P^{*}=\sum_{k=1}^{n}P_{k}.$$

It follows from Equations (1) and (3) that, with social interactions, a pooled record consists of the *A*_D_ and *E*_D_ of each group member, as well as their *A*_I_ and *E*_I_ that are expressed *n* – 1 times:

(4) $$P^{*}=\sum_{k=1}^{n}\left[A_{D_{k}}+E_{D_{k}}+\left(n-1\right)\;\left(A_{I_{k}}+E_{I_{k}}\right)\right].$$

Because an animal’s *A*_D_ and *A*_I_ are expressed in the same pooled record, the direct **Z**-matrix that links pooled phenotypes to *A*_D_’s and the indirect **Z**-matrix that links pooled phenotypes to *A*_I_’s are completely confounded (as shown in Appendix A by using a fictive example (Table 8)). Consequently, direct and indirect (co)variances, and breeding values cannot be estimated from pooled data.

It follows from Equations (2) and (4) that, with social interactions, a pooled record contains the total genetic effect of each group member:

(5) $$P^{*}=\sum_{k=1}^{n}\left(A_{T_{k}}+E_{k}\right).$$

Equation (5) shows strong similarities with:

(6) $$P^{*}=\sum_{k=1}^{n}\left(A_{D_{k}}+E_{k}\right),$$

which shows the content of a pooled record when social interactions do not occur. Previous studies have shown that pooled data can be used to estimate direct genetic variances ($\sigma_{A_{D}}^{2}$) and direct breeding values for traits that are not affected by social interactions [15-17]. Similarly, pooled data can be used to estimate total genetic variances ($\sigma_{A_{T}}^{2}$) and total breeding values for traits that are affected by social interactions.

#### Optimal group composition

In this section, the standard error (s.e.) of ${\hat{\sigma }}_{A_{T}}^{2}$ is derived for three experimental designs that differ with respect to group composition, *i.e.* group members belonged to either one, two or *n* families. The s.e. of an estimate of the genetic variance depends on the between- $\left(\sigma_{b}^{2}\right)\;$ and within-family variance $\left(\sigma_{w}^{2}\right)$, the relatedness within a family (*r*), the number of families (*N*), and the number of records per family (*m*) [18]:

(7) $$s.e.\left({\hat{\sigma }}_{A}^{2}\right)\approx \frac{1}{r}\sqrt{\frac{2}{N‒1}\left[\sigma_{b}^{4}+\frac{2\sigma_{b}^{2}\sigma_{w}^{2}}{m}+\frac{\sigma_{w}^{4}}{m\left(m‒1\right)}\right]}.$$

Analysis of variance was used to derive $\sigma_{b}^{2}$ and $\sigma_{w}^{2}$ for each design (see Appendix B for derivation).

The s.e. of ${\hat{\sigma }}_{A_{T}}^{2}$ differs between experimental designs because the group composition changes the within-family variance and the number of records per family (Table 2). On the one hand, the within-family variance decreases when the number of families per group decreases, causing a strong decrease in s.e.. On the other hand, the number of records per family decreases when the number of families per group decreases, causing a slight increase in s.e.. Overall, to obtain the most accurate estimate of $\sigma_{A_{T}}^{2}$, group members should belong to the same family. The only exception is when family size (*o*) equals group size (*n*). In this case, there is only one record per family and $\sigma_{A_{T}}^{2}$ would not be estimable.

**Table 2:** **Within-family variance (**$\sigma_{w}^{2}$**) and number of records per family ( *m *) for three group compositions**

	$\sigma_{w}^{2}$	***m***
One family	$\frac{1}{n}\left(\sigma_{P_{D}}^{2}+2\left(n-1\right)\sigma_{P_{\mathrm{DI}}}+{\left(n-1\right)}^{2}\;\sigma_{P_{I}}^{2}+\left(n-1\right)r\sigma_{A_{T}}^{2}\right)-r\sigma_{A_{T}}^{2}$	*o/n*
Two families	$\frac{4}{n}\left(\sigma_{P_{D}}^{2}+2\left(n-1\right)\sigma_{P_{\mathrm{DI}}}+{\left(n-1\right)}^{2}\;\sigma_{P_{I}}^{2}+\left(\frac{n}{2}-1\right)r\sigma_{A_{T}}^{2}\right)-r\sigma_{A_{T}}^{2}$	*2o/n*
*n* families	$n\;\left(\sigma_{P_{D}}^{2}+2\left(n-1\right)\;\sigma_{P_{\mathrm{DI}}}+{\left(n-1\right)}^{2}\sigma_{P_{I}}^{2}\right)-r\sigma_{A_{T}}^{2}$	*o*

*r*, *N*, *n, o* and $\sigma_{b}^{2}$ do not differ between group compositions.

Ideally, group members should be full sibs rather than half sibs, since an increase in relatedness causes a decrease in the s.e. of ${\hat{\sigma }}_{A_{T}}^{2}$.

### Simulation

To validate the theoretical derivations, a simulation study was conducted in R v2.12.2 [19]. A base population of 500 sires and 500 dams was simulated. Each animal in the base population was assigned a direct and indirect breeding value, drawn from $N\left(\left[\;\begin{array}{c} 0\\ 0 \end{array}\right],\;\left[\begin{array}{cc} \sigma_{A_{D}}^{2} & \sigma_{A_{\mathrm{DI}}}\\ \sigma_{A_{\mathrm{DI}}} & \sigma_{A_{I}}^{2} \end{array}\right]\right)$. The $\sigma_{A_{D}}^{2}$ and $\sigma_{A_{I}}^{2}$ were set to 1.00, and $\sigma_{A_{\mathrm{DI}}}$ was set to −0.50, 0.00 or 0.50. Each sire was randomly mated to a single dam, resulting in 12 offspring per mating for a total of 6000 simulated offspring. For each offspring, direct and indirect breeding values were obtained as: $A_{D}=\frac{1}{2}A_{D_{S}}+\frac{1}{2}\;A_{D_{D}}+MS_{D}$ and $A_{I}=\frac{1}{2}A_{I_{S}}+\frac{1}{2}\;A_{I_{D}}+MS_{I}$, where the direct and indirect Mendelian sampling terms were drawn from $N\left(\left[\;\begin{array}{c} 0\\ 0 \end{array}\right],\;\frac{1}{2}\left[\begin{array}{cc} \sigma_{A_{D}}^{2} & \sigma_{A_{\mathrm{DI}}}\\ \sigma_{A_{\mathrm{DI}}} & \sigma_{A_{I}}^{2} \end{array}\right]\right)$. Each offspring was also assigned a direct and indirect environmental value, drawn from $N\left(\left[\begin{array}{c} 0\\ 0 \end{array}\right],\;\left[\begin{array}{cc} \sigma_{E_{D}}^{2} & \sigma_{E_{\mathrm{DI}}}\\ \sigma_{E_{\mathrm{DI}}} & \sigma_{E_{I}}^{2} \end{array}\right]\right)$. The $\sigma_{E_{D}}^{2}$ and $\sigma_{E_{I}}^{2}$ were set to 2.00, and $\sigma_{E_{\mathrm{DI}}}$ was set to −1.00, 0.00 or 1.00. Animals were placed in groups of four. Depending on the scenario, group members belonged to one, two or four families. Individual phenotypes were obtained by summing the direct and indirect genetic and environmental components according to Equation (1). Pooled records were obtained by summing individual phenotypes according to Equation (3). Seven scenarios were simulated, which differed in $\sigma_{A_{\mathrm{DI}}}$, $\sigma_{E_{\mathrm{DI}}}$ or group composition (Table 3). For each scenario, 100 replicates were produced.

**Table 3:** Scenarios used to simulate data

	**Scenario**^**§**^	$\sigma_{A_{\mathrm{DI}}}$	$\sigma_{E_{\mathrm{DI}}}$	**Group composition**
Reference scenario	1	0.00	0.00	Four families
Different $\sigma_{A_{\mathrm{DI}}}$	2	−0.50	0.00	Four families
	3	0.50	0.00	Four families
Different $\sigma_{E_{\mathrm{DI}}}$	4	0.00	−1.00	Four families
	5	0.00	1.00	Four families
Different group compositions	6	0.00	0.00	Two families
	7	0.00	0.00	One family

^§^$\sigma_{A_{D}}^{2}$ and $\sigma_{A_{I}}^{2}$ were set to 1.00; $\sigma_{E_{D}}^{2}$ and $\sigma_{E_{I}}^{2}$ were set to 2.00.

Based on the previous section, expectations are that the use of a direct–indirect animal model for pooled data will fail to differentiate between direct and indirect genetic effects, while the use of a traditional animal model for pooled data will yield estimates of $\sigma_{A_{T}}^{2}$. To validate these theoretical predictions, both models were run. First, the simulated pooled records were analysed with the following direct–indirect animal model in ASReml v3.0 [20]:

(8) $$y^{*}=\mu^{*}+Z_{D}^{*}a_{D}+Z_{I}^{*}a_{I}+e^{*},$$

where y^*^ is a vector that contains pooled records (P^*^); μ^*^ is a vector that contains the pooled mean; $\;Z_{D}^{*}$ is an incidence matrix linking the pooled records to *A*_D_’s (each pooled record was linked to the *A*_D_’s of the four group members); **a**_D_ is a vector that contains *A*_D_’s; $Z_{I}^{*}$ is an incidence matrix linking the pooled records to *A*_I_’s (each pooled record was linked to the *A*_I_’s of the four group members); **a**_I_ is a vector that contains *A*_I_’s; and e^*^ is a vector that contains residuals. Second, the simulated pooled records were analysed with the following traditional animal model in ASReml v3.0 [20]:

(9) $$y^{*}=\mu^{*}+Z^{*}a+e^{*},$$

where y^*^, μ^*^ and e^*^ are as explained above; Z^*^ is an incidence matrix linking the pooled records to *A*’s (each pooled record was linked to the *A*’s of the four group members); and **a** is a vector that contains *A*’s.

Based on the previous section, expectations are that the most accurate prediction of $\sigma_{A_{T}}^{2}$ will be obtained when group members belong to the same family. To validate this theoretical prediction, the predicted s.e. of ${\hat{\sigma }}_{A_{T}}^{2}$ was compared to (i) the standard deviation (s.d.) of 100 estimates of $\sigma_{A_{T}}^{2}$ (${\hat{\sigma }}_{A_{T}}^{2}$’s reported by ASReml) and (ii) the mean of 100 s.e.’s of ${\hat{\sigma }}_{A_{T}}^{2}$ (s.e.’s reported by ASReml) for three group compositions (scenarios 1, 6 and 7 of Table 3).

### Data analyses

The dataset was part of the pre-existing database of Hendrix Genetics (The Netherlands) and contained routinely collected data for breeding value estimation. Animal Care and Use Committee approval was therefore not required.

To validate the theoretical derivations and to gain insight into response to selection, individual and pooled data on survival in purebred laying hens (*Gallus gallus*) were analysed. Survival in group-housed laying hens is a well-known example of a trait affected by social interactions, since a bird’s chance to survive depends on the feather pecking and cannibalistic behaviour of its group mates. Ellen et al. [5] used individual survival data on three purebred lines to estimate direct and indirect genetic (co)variances. Large and statistically significant indirect genetic effects were found in two out of three purebred lines. In the current study, we used data from the same two lines. Data were provided by the “Institut de Sélection Animale B.V.”, the layer breeding division of Hendrix Genetics. Data on 13 192 White Leghorn layers were provided of which 6276 were of line W1 and 6916 were of line WB.

At the age of 17 weeks, the hens were placed in two laying houses. The laying houses consisted of four or five double rows, and each row consisted of three levels. Interaction with neighbours on the back of the cage was possible, but interaction with neighbours on the side was prevented. Four hens of the same purebred line were randomly assigned to each cage. Hens were not beak-trimmed. Further details on housing conditions and management are in Ellen et al. [5].

The individual phenotype was defined as the number of days from the start of the laying period until either death or the end of the experiment, with a maximum of 398 days. The individual phenotypes were summed per cage to obtain pooled records. If one individual phenotype was missing, the entire cage was omitted from the analysis. The final dataset contained records on 6092 W1 and 6852 WB hens.

To obtain the direct, indirect and total genetic parameters for survival time, the individual phenotypes were analysed with the following direct–indirect animal model in ASReml v3.0 [20]:

(10) $$y=\mathrm{Xb}+Z_{D}a_{D}+Z_{I}a_{I}+\mathrm{Vcage}+e,$$

where **y** is a vector that contains individual phenotypes; **X** is an incidence matrix linking the individual phenotypes to fixed effects; **b** is a vector that contains fixed effects, which included an interaction term for each laying house by row by level combination, an effect for the content of the back cage (full/empty) and a covariate for the average number of survival days in the back cage; **Z**_D_ is an incidence matrix linking the individual phenotypes to *A*_D_’s; **a**_D_ is a vector that contains *A*_D_’s; **Z**_I_ is an incidence matrix linking the individual phenotypes to *A*_I_’s; **a**_I_ is a vector that contains *A*_I_’s; **V** is an incidence matrix linking the individual phenotypes to random cage effects; **cage** is a vector that contains random cage effects (to account for the non-genetic covariance among phenotypes of cage members [21]); and **e** is a vector that contains residuals. This model yields estimates of $\sigma_{A_{D}}^{2}$, $\sigma_{A_{\mathrm{DI}}}$ and $\sigma_{A_{I}}^{2}$, from which ${\hat{\sigma }}_{A_{T}}^{2}$ can be calculated. Similarly, it yields estimates of *A*_D_’s and *A*_I_’s, from which ${\hat{A}}_{T}$’s can be calculated. To improve a trait, animals should be selected based on their ${\hat{A}}_{T}$, since $\sigma_{A_{T}}^{2}$ determines a population’s potential to respond to selection.

Alternatively, a traditional animal model can be used to analyse individual or pooled data. A traditional animal model on individual data only yields estimates of $\sigma_{A_{D}}^{2}$ and *A*_D_’s. A traditional model on pooled data is expected to yield estimates of $\sigma_{A_{T}}^{2}$ and *A*_T_’s, but not of $\sigma_{A_{D}}^{2}$ and *A*_D_’s. To validate this theoretical prediction, these traditional models were also run. First, the individual phenotypes were analysed with the following traditional (direct) animal model in ASReml v3.0 [20]:

(11) $$y=\mathrm{Xb}+Z_{D}a_{D}+\mathrm{Vcage}+e,$$

where **y**, **X**, **b**, **Z**_D_, **a**_D_, **V**, **cage** and **e** are as explained above. Second, the pooled records were analysed with the following traditional animal model in ASReml v3.0 [20]:

(12) $$y^{*}=X^{*}b^{*}+Z^{*}a+e^{*},$$

where y^*^ is a vector that contains pooled records (P^*^); X^*^ is an incidence matrix linking the pooled records to fixed effects; b^*^ is a vector that contains fixed effects (the same fixed effects as mentioned above); Z^*^ is an incidence matrix linking the pooled records to *A*’s (each pooled record was linked to the *A*’s of the four group members); **a** is a vector that contains *A*’s; and e^*^ is a vector that contains residuals.

The estimated variance components and breeding values of all three models were compared. In addition, we calculated the loss in response to selection that would occur when applying a traditional model to individual or pooled data instead of a direct–indirect model to individual data. The direct–indirect model applied to individual data yielded estimates of $\sigma_{A_{T}}^{2}$ and *A*_T_’s. Based on their ${\hat{A}}_{T}$, 250 animals were selected and the corresponding response to selection was calculated. Similarly, for the two traditional animal models, 250 animals were selected based on their ${\hat{A}}_{D}$ (obtained from individual data) and $\hat{A}$ (obtained from pooled data). Once the top 250 animals were selected, their ${\hat{A}}_{T}$ (obtained from individual data) was used to calculate the total response to selection. Then, the loss in total response to selection was calculated.

## Results and discussion

### Simulation

The direct–indirect animal model on pooled records failed to converge, confirming that direct and indirect (co)variances cannot be estimated from pooled data. The traditional animal model on pooled records yielded estimates of $\sigma_{A}^{2}$ and $\sigma_{E^{*}}^{2}$. These estimates did not differ significantly from the true $\sigma_{A_{T}}^{2}$ and $\sigma_{E^{*}}^{2}$ (Table 4), where

(13) $$\sigma_{A_{T}}^{2}=\sigma_{A_{D}}^{2}+2\left(n-1\right){\;\sigma }_{A_{\mathrm{DI}}}+{\left(n-1\right)}^{2}{\;\sigma }_{A_{I}}^{2}$$

(derived by [14]) and

(14) $$\sigma_{E^{*}}^{2}=n\left[\sigma_{E_{D}}^{2}+2\left(n-1\right)\;\sigma_{E_{\mathrm{DI}}}+{\left(n-1\right)}^{2}\sigma_{E_{I}}^{2}\right]$$

(analogous to [17]).

**Table 4:** **True and estimated**$\sigma_{A_{T}}^{2}$**and**$\sigma_{E^{*}}^{2}$**for five scenarios**

	**Scenario**^**§**^	$\sigma_{A_{T}}^{2}$ ^**§§**^	$\bar{{\hat{\sigma }}_{A}^{2}\pm s.e.}$	$\sigma_{E^{*}}^{2}$ ^**§§§**^	$\bar{{\hat{\sigma }}_{E^{*}}^{2}\pm s.e.}$
$\sigma_{A_{\mathrm{DI}}}=0.00$	1	10.00	10.10 ± 1.85	80.00	80.56 ± 6.69
$$\sigma_{E_{\mathrm{DI}}}=0.00$$					
$\sigma_{A_{\mathrm{DI}}}=-0.50$	2	7.00	7.43 ± 1.59	80.00	79.29 ± 6.08
$$\sigma_{E_{\mathrm{DI}}}=0.00$$					
$\sigma_{A_{\mathrm{DI}}}=0.50$	3	13.00	13.05 ± 2.12	80.00	80.32 ± 7.30
$$\sigma_{E_{\mathrm{DI}}}=0.00$$					
$\sigma_{A_{\mathrm{DI}}}=0.00$	4	10.00	9.70 ± 1.54	56.00	56.54 ± 5.24
$$\sigma_{E_{\mathrm{DI}}}=-1.00$$					
$\sigma_{A_{\mathrm{DI}}}=0.00$	5	10.00	9.81 ± 2.10	104.00	104.71 ± 8.03
$$\sigma_{E_{\mathrm{DI}}}=1.00$$					

^§^$\sigma_{A_{D}}^{2}$ and $\sigma_{A_{I}}^{2}$ were set to 1.00; $\sigma_{E_{D}}^{2}$ and $\sigma_{E_{I}}^{2}$ were set to 2.00; group members belonged to four different families.

^§§^$\sigma_{A_{T}}^{2}=\sigma_{A_{D}}^{2}+2\left(n-1\right)\;\sigma_{A_{\mathrm{DI}}}+{\left(n-1\right)}^{2}\;\sigma_{A_{I}}^{2}$.

^§§§^$\sigma_{E^{*}}^{2}=n\left[\sigma_{E_{D}}^{2}+2\left(n-1\right)\;\sigma_{E_{\mathrm{DI}}}+{\left(n-1\right)}^{2}\;\sigma_{E_{I}}^{2}\right]$.

Based on Equation (7), the s.e. of ${\hat{\sigma }}_{A_{T}}^{2}$ was predicted for three scenarios that differed in group composition, *i.e.* group members belonged to one, two or four families. The theoretical s.e. of ${\hat{\sigma }}_{A_{T}}^{2}$ was compared to (i) the s.d. of 100 estimates of $\sigma_{A_{T}}^{2}$ (${\hat{\sigma }}_{A_{T}}^{2}$’s reported by ASReml) and (ii) the mean of 100 s.e.’s of ${\hat{\sigma }}_{A_{T}}^{2}$ (s.e.’s reported by ASReml) (Table 5). The theoretical s.e. of ${\hat{\sigma }}_{A_{T}}^{2}$ did not differ significantly from the values obtained by simulation. Moreover, as predicted, the most accurate estimate of $\sigma_{A_{T}}^{2}$ was obtained when group members belonged to the same family. In comparison, the s.e. of ${\hat{\sigma }}_{A_{T}}^{2}$ was twice as large when group members belonged to different families. This indicates that group composition is crucial when aiming to obtain accurate estimates.

**Table 5:** **Theoretically predicted**$s.e.\left({\hat{\sigma }}_{A_{T}}^{2}\right)$**,**$s.d.\left({\hat{\sigma }}_{A_{T}}^{2}\right)$^**§ **^**and**$\bar{s.e.\left({\hat{\sigma }}_{A_{T}}^{2}\right)}$^**§§ **^**for three group compositions**

	**Scenario**^**§§§**^	$s.e.\left({\hat{\sigma }}_{A_{T}}^{2}\right)$	$s.d.\left({\hat{\sigma }}_{A_{T}}^{2}\right)\pm s.d.$	$\bar{s.e.\left({\hat{\sigma }}_{A_{T}}^{2}\right)}\pm s.d.$
Four families	1	1.88	2.01 ± 0.14	1.85 ± 0.13
Two families	6	1.30	1.23 ± 0.09	1.23 ± 0.08
One family	7	0.92	0.81 ± 0.06	0.92 ± 0.05

^§^$s.d.\left({\hat{\sigma }}_{A_{T}}^{2}\right)$ based on 100 ${\hat{\sigma }}_{A_{T}}^{2}$’s reported by ASReml.

^§§^$\bar{s.e.\left({\hat{\sigma }}_{A_{T}}^{2}\right)}$ based on 100 s.e.’s reported by ASReml.

^§§§^$\sigma_{A_{D}}^{2}$ and $\sigma_{A_{I}}^{2}$ were set to 1.00; $\sigma_{A_{\mathrm{DI}}}$ was set to 0.00; $\sigma_{E_{D}}^{2}$ and $\sigma_{E_{I}}^{2}$ were set to 2.00; $\sigma_{E_{\mathrm{DI}}}$ was set to 0.00.

### Data analyses

Table 6 shows the estimated variance components for individual survival data analysed with a direct–indirect animal model, and the estimated variance components for individual and pooled survival data analysed with a traditional animal model. The direct–indirect animal model on individual data yielded estimates of $\sigma_{A_{D}}^{2}$, ${\;\sigma }_{A_{\mathrm{DI}}}$ and $\sigma_{A_{I}}^{2}$. Based on these components, ${\hat{\sigma }}_{A_{T}}^{2}$ was calculated (according to Equation (13)). The traditional animal model on individual data yielded estimates of $\sigma_{A_{D}}^{2}$. The traditional animal model on pooled data yielded estimates of $\sigma_{A}^{2}$ that closely resembled the estimates of $\sigma_{A_{T}}^{2}$ from individual data. The direct–indirect animal model on individual data also yielded estimates of $\sigma_{\mathrm{Cage}}^{2}$ and $\sigma_{E}^{2}$. As derived by Bergsma et al. [21], ${\hat{\sigma }}_{\mathrm{Cage}}^{2}$ is an estimate of $2\sigma_{E_{\mathrm{DI}}}+\left(n-2\right)\sigma_{E_{I}}^{2}$. As derived by Bijma [22], ${\hat{\sigma }}_{E}^{2}$ is an estimate of $\sigma_{E_{D}}^{2}-2\sigma_{E_{\mathrm{DI}}}+\sigma_{E_{I}}^{2}$. As shown in Equation (14), ${\hat{\sigma }}_{E^{*}}^{2}$ is an estimate of $n\left[\sigma_{E_{D}}^{2}+2\left(n-1\right){\;\sigma }_{E_{\mathrm{DI}}}+{\left(n-1\right)}^{2}{\;\sigma }_{E_{I}}^{2}\right]$. Consequently, the ${\hat{\sigma }}_{\mathrm{Cage}}^{2}$ and ${\hat{\sigma }}_{E}^{2}$ from the direct–indirect animal model on individual data should sum to the ${\hat{\sigma }}_{E^{*}}^{2}$ from the traditional animal model on pooled data. More precisely:

(15) $${\hat{\sigma }}_{E^{*}}^{2}=n^{2}{\hat{\sigma }}_{\mathrm{Cage}}^{2}+n{\hat{\sigma }}_{E}^{2}.$$

**Table 6:** Estimated variance components (with s.e.) from individual and pooled data on survival in laying hens

	**W1**	**WB**
Direct–indirect animal model on individual data		
$\sigma_{A_{D}}^{2}$	705 (± 171)	1404 (± 301)
$\sigma_{A_{\mathrm{DI}}}$	59 (± 61)	−162 (± 105)
$\sigma_{A_{I}}^{2}$	104 (± 41)	232 (± 72)
$\sigma_{\mathrm{Cage}}^{2}$	799 (± 166)	1191 (± 238)
$\sigma_{E}^{2}$	7980 (± 210)	12 675 (± 365)
$\sigma_{A_{T}}^{2}$ ^§^	1996 (± 640)	2521 (± 842)
Expected $\sigma_{E^{*}}^{2}$^§§^	44 700 (± 2526)	69 752 (± 3513)
Traditional (direct) animal model on individual data		
$\sigma_{A_{D}}^{2}$	677 (± 165)	1522 (± 317)
$\sigma_{\mathrm{Cage}}^{2}$	1096 (± 127)	1443 (± 186)
$\sigma_{E}^{2}$	8002 (± 205)	13 008 (± 338)
Traditional animal model on pooled data		
$\sigma_{A}^{2}$	1979 (± 643)	2521 (± 845)
$\sigma_{E^{*}}^{2}$	44 750 (± 2538)	69 750 (± 3519)

^§^ In groups of four, $\sigma_{A_{T}}^{2}$ equals $\sigma_{A_{D}}^{2}+6{\;\sigma }_{A_{\mathrm{DI}}}+9{\;\sigma }_{A_{I}}^{2}$.

^§§^ In groups of four, $\sigma_{E^{*}}^{2}$ equals $16{\;\sigma }_{\mathrm{Cage}}^{2}+4{\;\sigma }_{E}^{2}$.

The expected ${\hat{\sigma }}_{E^{*}}^{2}$, calculated based on the ${\hat{\sigma }}_{\mathrm{Cage}}^{2}$ and ${\hat{\sigma }}_{E}^{2}$ from the direct–indirect animal model on individual data, and the ${\hat{\sigma }}_{E^{*}}^{2}$ from the traditional animal model on pooled data closely resembled each other.

Table 6 does not show heritability estimates. Where the classical heritability (*h*^2^) is used to express $\sigma_{A_{D}}^{2}$ relative to the phenotypic variance ($\sigma_{P}^{2}$), *T*^*2*^ is used to express $\sigma_{A_{T}}^{2}$ relative to $\sigma_{P}^{2}$[21]. Comparing values of *T*^*2*^ obtained from individual and pooled data would be misleading because they are not expected to be similar. Unlike for a trait that is not affected by social interactions, $\sigma_{P^{*}}^{2}$ cannot simply be divided by the number of group members to obtain $\sigma_{P}^{2}$. When group members are unrelated,

(16) $$\sigma_{P}^{2}=\sigma_{A_{D}}^{2}+\left(n-1\right)\sigma_{A_{I}}^{2}+\sigma_{E_{D}}^{2}+\left(n-1\right)\sigma_{E_{I}}^{2}$$

and

(17) $$\begin{array}{l} \sigma_{P^{*}}^{2}={\mathrm{n\sigma}}_{A_{T}}^{2}+\sigma_{E^{*}}^{2}\\ \;=n[\sigma_{A_{D}}^{2}+2\left(n-1\right)\sigma_{A_{\mathrm{DI}}}+{\left(n-1\right)}^{2}\sigma_{A_{I}}^{2}\\ \;+\sigma_{E_{D}}^{2}+2\left(n-1\right)\sigma_{E_{\mathrm{DI}}}+{\left(n-1\right)}^{2}\sigma_{E_{I}}^{2}]. \end{array}$$

The non-proportional increase of $\sigma_{P}^{2}$ does not enable a meaningful comparison between values of *T*^*2*^ obtained from individual and pooled data.

In conclusion, when group members are unrelated, a traditional animal model on individual data yields estimates of $\sigma_{A_{D}}^{2}$, while a traditional animal model on pooled data yields estimates of $\sigma_{A_{T}}^{2}$. Moreover, the estimated cage and error variances from a direct–indirect animal model on individual data sum to the pooled error variance from a traditional animal model on pooled data. This result could explain the ‘inconsistencies’ found by Biscarini et al. [17], who assumed that a traditional animal model on individual and pooled data should yield the same genetic variance. Moreover, Biscarini et al. [17] expected to find a pooled error variance that is four times larger than the individual error variance. For body weight at the age of 19 and 27 weeks, these expectations were met. For body weight at the age of 43 and 51 weeks, however, the genetic variance estimated from pooled data was smaller than expected, while the pooled error variance was larger than expected. Biscarini et al. [17] mentions the emergence of competition effects as a possible cause. We indeed expect to find indirect genetic effects when the individual data on body weight at the age of 43 and 51 weeks were reanalysed with a direct–indirect animal model. Using Equations (13) and (15), the estimated variance components from individual data would resemble the estimated variance components from pooled data.

The regression coefficients of ${\hat{A}}_{D}$’s obtained from individual data on the $\hat{A}$’s obtained from pooled data strongly deviated from one (0.363 ± 0.006 for W1; 0.392 ± 0.010 for WB). The regression coefficients of ${\hat{A}}_{T}$’s obtained from individual data on the $\hat{A}$’s obtained from pooled data were close to, and not significantly different from, one (1.004 ± 0.003 for W1; 1.001 ± 0.001 for WB). This indicates that the $\hat{A}$’s obtained from pooled data are unbiased estimates of the ${\hat{A}}_{T}$’s obtained from individual data.

Table 7 shows Spearman correlation coefficients between ${\hat{A}}_{D}$’s and ${\hat{A}}_{T}$’s obtained from individual data and the $\hat{A}$’s obtained from pooled data. The Spearman correlation coefficients between the ${\hat{A}}_{T}$’s obtained from individual data and the $\hat{A}$’s obtained from pooled data were close to, but significantly different from, one. This indicates only a minor loss in the accuracy of ${\hat{A}}_{T}$’s when using pooled instead of individual data, which will be reflected in a minor loss in response to selection when using pooled instead of individual data.

**Table 7:** **Spearman correlation coefficients between**${\hat{A}}_{D}$**’s and**${\hat{A}}_{T}$**’s obtained from individual data and**$\hat{A}$**’s from pooled data on survival in laying hens**

	${\hat{A}}_{D}$	${\hat{A}}_{T}$	$\hat{A}$
${\hat{A}}_{D}$		0.513 (± 0.001)	0.412 (± 0.001)
${\hat{A}}_{T}$	0.725 (± 0.001)		0.992 (± 0.001)
$\hat{A}$	0.543 (± 0.001)	0.967 (± 0.001)	

Spearman correlation coefficients for data on W1 hens below the diagonal and for data on WB hens above the diagonal.

**Figure 1:**
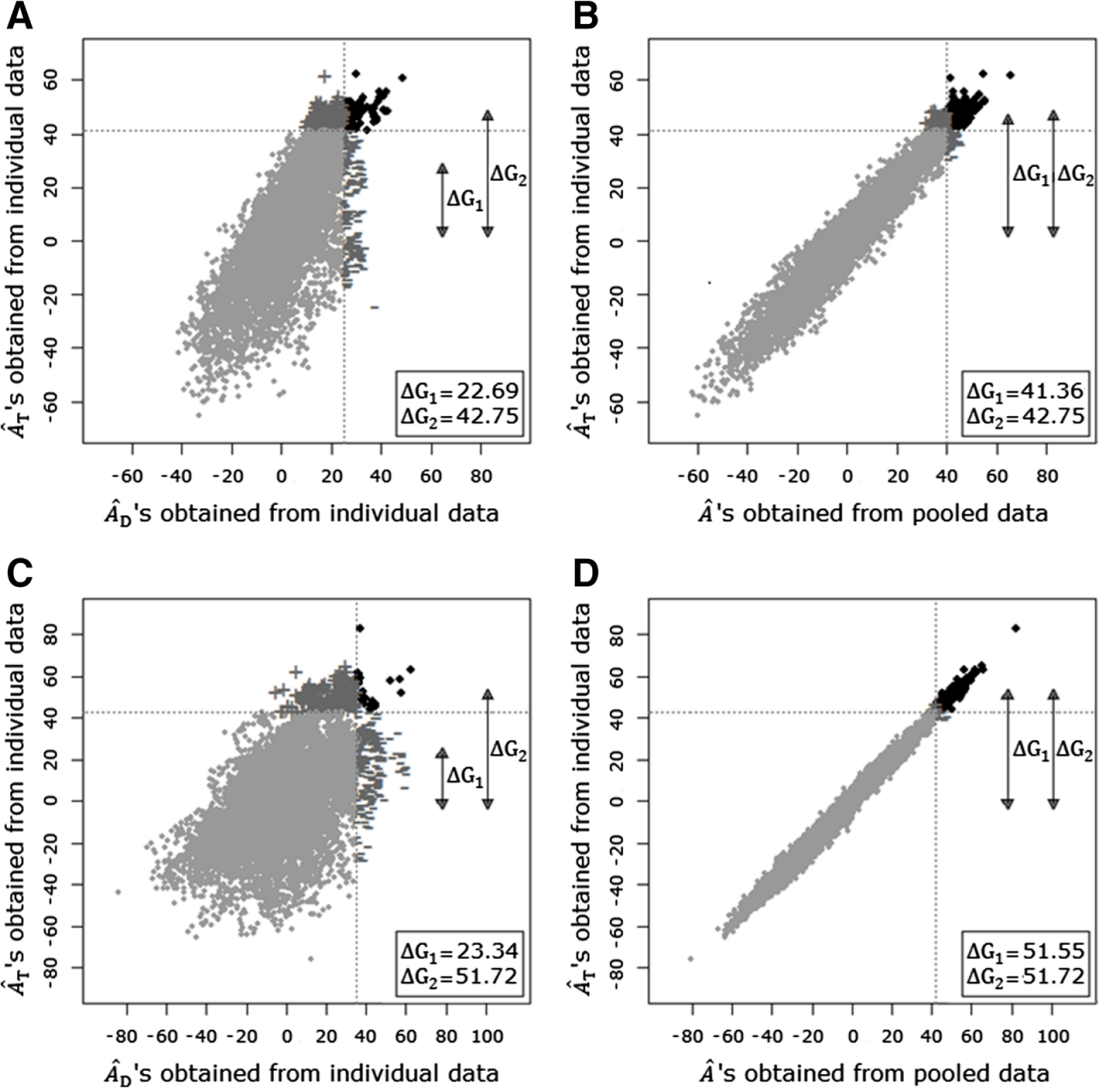
$${\hat{A}}_{T}$$**’s obtained from individual data plotted against**${\hat{A}}_{D}$**’s obtained from individual data and**$\hat{A}$**’s obtained from pooled data on survival in laying hens. A** and **B** for data on W1 hens. **C** and **D** for data on WB hens. ∆G_1_ represents the total response to selection when selecting animals based on their ${\hat{A}}_{D}$ obtained from individual data or $\hat{A}$ obtained from pooled data. ∆G_2_ represents the total response to selection when selecting animals based on their ${\hat{A}}_{T}$ obtained from individual data.

To gain more insight, we calculated the loss in response to selection that occurs when applying a traditional model to individual or pooled data instead of a direct–indirect model to individual data. When applying a traditional model to individual data, the loss in total response to selection was 46.9% for W1 (Figure 1A) and 54.9% for WB (Figure 1C). When applying a traditional model to pooled data, the loss in total response to selection was 3.3% for W1 (Figure 1B) and 0.3% for WB (Figure 1D). In conclusion, the loss in total response to selection will be large when using a traditional animal model on individual data, but will be small when using a traditional animal model on pooled data. However, this outcome may be specific to this dataset. Survival in purebred laying hens was recorded in cages with four unrelated birds. Both direct and indirect genetic effects strongly influenced the trait. Group size, group composition, and the relative impact of direct and indirect genetic effects might influence the loss in total response to selection. For example, for body weight at 19 and 27 weeks of age, indirect genetic effects are expected to be small. In that case, an animal’s *A*_T_ is mainly expressed in the phenotype of the animal itself. Consequently, we expect that more accurate estimated breeding values can be obtained when using individual instead of pooled data. Biscarini et al. [17] found a correlation of ~ 0.75 between the estimated breeding values based on individual and pooled data, resulting in a large loss in response to selection when using pooled instead of individual data. Thus, using pooled data does not always seem to be a proper alternative and requires further research.

## Conclusions

Using pooled data, the total genetic variance and breeding values can be estimated, but the underlying direct and indirect genetic (co)variances and breeding values cannot. The most accurate estimates are obtained when group members belong to the same family. While quantifying the direct and indirect genetic effects is interesting from a biological perspective, obtaining the total genetic effect is most important from an animal breeding perspective. When it is too difficult or expensive to obtain individual data, pooled data can be used to improve traits.

## Appendix A

This section demonstrates why direct and indirect (co)variances can be estimated from individual data, but cannot be estimated from pooled data.

Consider a situation where four base parents produce six offspring. Animals are kept in groups of two and individual phenotypes are recorded on all six offspring (Table 8).

**Table 8:** Example pedigree structure and group composition

**Animal**	**Sire**	**Dam**	**Phenotype**	**Group**
1	-	-	-	-
2	-	-	-	-
3	-	-	-	-
4	-	-	-	-
5	1	3	✓	1
6	2	4	✓	1
7	1	4	✓	2
8	2	3	✓	2
9	2	3	✓	3
10	2	4	✓	3

When analysing individual data with a direct–indirect animal model, the **Z**-matrices would be:

$$\begin{array}{c} Z_{D}=\left[\begin{array}{c} \begin{array}{ccc} 0 & 0 & \begin{array}{ccc} 0 & 0 & \begin{array}{ccc} 1 & 0 & \begin{array}{ccc} 0 & 0 & \begin{array}{cc} 0 & 0 \end{array} \end{array} \end{array} \end{array} \end{array}\\ \begin{array}{ccc} 0 & 0 & \begin{array}{ccc} 0 & 0 & \begin{array}{ccc} 0 & 1 & \begin{array}{ccc} 0 & 0 & \begin{array}{cc} 0 & 0 \end{array} \end{array} \end{array} \end{array} \end{array}\\ \begin{array}{ccc} \begin{array}{c} 0\\ 0\\ \begin{array}{c} 0\\ 0 \end{array} \end{array} & \begin{array}{c} 0\\ 0\\ \begin{array}{c} 0\\ 0 \end{array} \end{array} & \begin{array}{ccc} \begin{array}{c} 0\\ 0\\ \begin{array}{c} 0\\ 0 \end{array} \end{array} & \begin{array}{c} 0\\ 0\\ \begin{array}{c} 0\\ 0 \end{array} \end{array} & \begin{array}{ccc} \begin{array}{c} 0\\ 0\\ \begin{array}{c} 0\\ 0 \end{array} \end{array} & \begin{array}{c} 0\\ 0\\ \begin{array}{c} 0\\ 0 \end{array} \end{array} & \begin{array}{cc} \begin{array}{c} 1\\ 0\\ \begin{array}{c} 0\\ 0 \end{array} \end{array} & \begin{array}{cc} \begin{array}{c} 0\\ 1\\ \begin{array}{c} 0\\ 0 \end{array} \end{array} & \begin{array}{cc} \begin{array}{c} 0\\ 0\\ \begin{array}{c} 1\\ 0 \end{array} \end{array} & \begin{array}{c} 0\\ 0\\ \begin{array}{c} 0\\ 1 \end{array} \end{array} \end{array} \end{array} \end{array} \end{array} \end{array} \end{array} \end{array}\right],\\ Z_{I}=\left[\begin{array}{c} \begin{array}{ccc} 0 & 0 & \begin{array}{ccc} 0 & 0 & \begin{array}{ccc} 0 & 1 & \begin{array}{ccc} 0 & 0 & \begin{array}{cc} 0 & 0 \end{array} \end{array} \end{array} \end{array} \end{array}\\ \begin{array}{ccc} 0 & 0 & \begin{array}{ccc} 0 & 0 & \begin{array}{ccc} 1 & 0 & \begin{array}{ccc} 0 & 0 & \begin{array}{cc} 0 & 0 \end{array} \end{array} \end{array} \end{array} \end{array}\\ \begin{array}{ccc} \begin{array}{c} 0\\ 0\\ \begin{array}{c} 0\\ 0 \end{array} \end{array} & \begin{array}{c} 0\\ 0\\ \begin{array}{c} 0\\ 0 \end{array} \end{array} & \begin{array}{ccc} \begin{array}{c} 0\\ 0\\ \begin{array}{c} 0\\ 0 \end{array} \end{array} & \begin{array}{c} 0\\ 0\\ \begin{array}{c} 0\\ 0 \end{array} \end{array} & \begin{array}{ccc} \begin{array}{c} 0\\ 0\\ \begin{array}{c} 0\\ 0 \end{array} \end{array} & \begin{array}{c} 0\\ 0\\ \begin{array}{c} 0\\ 0 \end{array} \end{array} & \begin{array}{cc} \begin{array}{c} 0\\ 1\\ \begin{array}{c} 0\\ 0 \end{array} \end{array} & \begin{array}{cc} \begin{array}{c} 1\\ 0\\ \begin{array}{c} 0\\ 0 \end{array} \end{array} & \begin{array}{cc} \begin{array}{c} 0\\ 0\\ \begin{array}{c} 0\\ 1 \end{array} \end{array} & \begin{array}{c} 0\\ 0\\ \begin{array}{c} 1\\ 0 \end{array} \end{array} \end{array} \end{array} \end{array} \end{array} \end{array} \end{array} \end{array}\right]. \end{array}$$

**Z**_**D**_ and **Z**_**I**_ are not identical, indicating that the direct and indirect genetic effects are estimated based on different information sources, enabling the model to distinguish between these two effects.

When analysing pooled data with a direct–indirect animal model, the **Z**-matrices would be:

$$\begin{array}{c} Z_{D}^{*}=\left[\begin{array}{c} \begin{array}{ccc} 0 & 0 & \begin{array}{ccc} 0 & 0 & \begin{array}{ccc} 1 & 1 & \begin{array}{ccc} 0 & 0 & \begin{array}{cc} 0 & 0 \end{array} \end{array} \end{array} \end{array} \end{array}\\ \begin{array}{ccc} 0 & 0 & \begin{array}{ccc} 0 & 0 & \begin{array}{ccc} 0 & 0 & \begin{array}{ccc} 1 & 1 & \begin{array}{cc} 0 & 0 \end{array} \end{array} \end{array} \end{array} \end{array}\\ \begin{array}{ccc} 0 & 0 & \begin{array}{ccc} 0 & 0 & \begin{array}{ccc} 0 & 0 & \begin{array}{cc} 0 & \begin{array}{cc} 0 & \begin{array}{cc} 1 & 1 \end{array} \end{array} \end{array} \end{array} \end{array} \end{array} \end{array}\right],\\ Z_{I}^{*}=\left[\begin{array}{c} \begin{array}{ccc} 0 & 0 & \begin{array}{ccc} 0 & 0 & \begin{array}{ccc} 1 & 1 & \begin{array}{ccc} 0 & 0 & \begin{array}{cc} 0 & 0 \end{array} \end{array} \end{array} \end{array} \end{array}\\ \begin{array}{ccc} 0 & 0 & \begin{array}{ccc} 0 & 0 & \begin{array}{ccc} 0 & 0 & \begin{array}{ccc} 1 & 1 & \begin{array}{cc} 0 & 0 \end{array} \end{array} \end{array} \end{array} \end{array}\\ \begin{array}{ccc} 0 & 0 & \begin{array}{ccc} 0 & 0 & \begin{array}{ccc} 0 & 0 & \begin{array}{cc} 0 & \begin{array}{cc} 0 & \begin{array}{cc} 1 & 1 \end{array} \end{array} \end{array} \end{array} \end{array} \end{array} \end{array}\right]. \end{array}$$

$Z_{D}^{*}$ and $Z_{I}^{*}$ are identical, indicating that the direct and indirect genetic effects are estimated based on the same information source, causing complete confounding between direct and indirect genetic effects. The model will not be able to distinguish between these two effects.

## Appendix B

Components of variance are determined by analysis of variance, where the full variance $\;\left(\sigma_{z}^{2}\right)$ is partitioned into a between- $\left(\sigma_{b}^{2}\right)\;$ and within-family component ($\sigma_{w}^{2}$). In this section, the derivation of $\sigma_{z}^{2}$, $\sigma_{b}^{2}$ and $\sigma_{w}^{2}$ are presented for three group compositions.

(i) When the group is composed of only one family, the *A*_T_ of a family is expressed *n* times in the same pooled record. Therefore, the record of interest is P^*^/*n*.

$$\begin{array}{l} \sigma_{z}^{2}=\frac{\sigma_{P^{*}}^{2}}{n^{2}}=\frac{n\;\left(\sigma_{P_{D}}^{2}+2\left(n-1\right)\sigma_{P_{\mathrm{DI}}}+{\left(n-1\right)}^{2}\;\sigma_{P_{I}}^{2}\right)\;}{n^{2}}\\ +\frac{n\left(n-1\right)r\;\left(\sigma_{A_{D}}^{2}+2\left(n-1\right)\;\sigma_{A_{\mathrm{DI}}}+{\left(n-1\right)}^{2}\;\sigma_{A_{I}}^{2}\right)}{n^{2}}\\ =\frac{1}{n}\left(\sigma_{P_{D}}^{2}+2\left(n-1\right)\sigma_{P_{\mathrm{DI}}}+{\left(n-1\right)}^{2}\;\sigma_{P_{I}}^{2}+\left(n-1\right)r\;\sigma_{A_{T}}^{2}\right)\\ \sigma_{b}^{2}=r\;\sigma_{A_{T}}^{2}\\ \sigma_{w}^{2}=\frac{1}{n}\left(\sigma_{P_{D}}^{2}+2\left(n-1\right)\sigma_{P_{\mathrm{DI}}}+{\left(n-1\right)}^{2}\sigma_{P_{I}}^{2}+\left(n-1\right)r\;\sigma_{A_{T}}^{2}\right)-r\;\sigma_{A_{T}}^{2} \end{array}$$

(ii) When the group is composed of two families, the *A*_T_ of a family is expressed *n*/2 times in the same pooled record. Therefore, the record of interest is 2P^*^/*n*.

$$\begin{array}{l} \sigma_{z}^{2}=\frac{4\sigma_{P^{*}}^{2}}{n^{2}}=\frac{4n\;\left(\sigma_{P_{D}}^{2}+2\left(n-1\right)\;\sigma_{P_{\mathrm{DI}}}+{\left(n-1\right)}^{2}\;\sigma_{P_{I}}^{2}\right)\;}{n^{2}}\\ +\frac{4n\left(\frac{n}{2}-1\right)r\;\left(\sigma_{A_{D}}^{2}+2\left(n-1\right)\;\sigma_{A_{\mathrm{DI}}}+{\left(n-1\right)}^{2}\;\sigma_{A_{I}}^{2}\right)}{n^{2}}\\ =\frac{4}{n}(\sigma_{P_{D}}^{2}+2\left(n-1\right)\sigma_{P_{\mathrm{DI}}}+{\left(n-1\right)}^{2}\;\sigma_{P_{I}}^{2}+\left(\frac{n}{2}-1\right)r\;\sigma_{A_{T}}^{2})\\ \sigma_{b}^{2}=r\;\sigma_{A_{T}}^{2}\\ \sigma_{w}^{2}=\frac{4}{n}\left(\sigma_{P_{D}}^{2}+2\left(n-1\right)\sigma_{P_{\mathrm{DI}}}+{\left(n-1\right)}^{2}\;\sigma_{P_{I}}^{2}\right)\\ \;\left(+\left(\frac{n}{2}-1\right)r\;\sigma_{A_{T}}^{2}\right)-r\;\sigma_{A_{T}}^{2} \end{array}$$

(iii) When the group composition is random, the *A*_T_ of a family is only expressed once per pooled record. Therefore, the record of interest is P^*^.

$$\begin{array}{l} \sigma_{z}^{2}=\sigma_{P^{*}}^{2}=n\;\left(\sigma_{P_{D}}^{2}+2\left(n-1\right)\;\sigma_{P_{\mathrm{DI}}}+{\left(n-1\right)}^{2}\;\sigma_{P_{I}}^{2}\right)\\ \sigma_{b}^{2}=r\;\sigma_{A_{T}}^{2}\\ \sigma_{w}^{2}=n\;\left(\sigma_{P_{D}}^{2}+2\left(n-1\right)\;\sigma_{P_{\mathrm{DI}}}+{\left(n-1\right)}^{2}\;\sigma_{P_{I}}^{2}\right)-r\;\sigma_{A_{T}}^{2} \end{array}$$

## Competing interests

The authors declare that they have no competing interests.

## Authors’ contributions

KP, EDE and PB participated in the design of the study. KP conducted the study. KP, EDE and PB wrote the paper. PB was the principal supervisor of the study. All authors read and approved the manuscript.
